# “Tell Me”: Disclosing Sexual Abuse by Survivors from the LGBTQ+ Community via Social Media

**DOI:** 10.3390/bs15040435

**Published:** 2025-03-28

**Authors:** Nofar Mazursky, Dafna Tener, Yochay Nadan, Ziv Aviram

**Affiliations:** 1 Marron Institute of Urban Management, New York University, 370 Jay Street, Brooklyn, New York, NY 11201, USA; 2 Center for Urban Science and Progress, New York University Tandon School of Engineering, 370 Jay Street, Brooklyn, New York, NY 11201, USA; 3 Paul Baerwald School of Social Work and Social Welfare, Hebrew University of Jerusalem, Mount Scopus, Jerusalem 9190501, Israel; dafna.tener@mail.huji.ac.il (D.T.); yochay.nadan@mail.huji.ac.il (Y.N.); ziv.aviram2@gmail.com (Z.A.)

**Keywords:** LGBTQ+ community, sexual abuse (SA), sexual abuse disclosure, social media, qualitative research, survivors, posts

## Abstract

(1) Background: Members of the LGBTQ+ community face specific risk factors that make them more vulnerable to multiple forms of sexual abuse and victimization than cisgender heterosexual individuals. Furthermore, numerous personal, intrapersonal, societal, and cultural factors inhibit disclosure, threatening their wellbeing. The purpose of the current study was to explore sexual abuse experiences among survivors from the LGBTQ+ community. (2) Methods: An analysis of sexual abuse disclosure posts published on an Israeli Instagram page called Torenu was conducted. Through the analysis of these posts, this study aimed to better understand the unique characteristics and dynamics of the sexual abuse of survivors from the LGBTQ+ community. Sixty-five posts from members of the LGBTQ+ community disclosing sexual abuse were analyzed using qualitative thematic analysis. (3) Results: Two themes emerged during the analysis. The first theme focused on the characteristics of the survivors and perpetrators. The second theme related to the abuse characteristics, addressing four main aspects: publicness, normalization, numbness of the senses, and escalation. (4) Conclusions: Understanding the nature of sexual abuse within the LGBTQ+ community could encourage society to take responsibility for fostering an inclusive discourse on sexuality, one that acknowledges LGBTQ+ identities, reduces stigma, promotes visibility, and facilitates intervention within the community.

## 1. Introduction

LGBTQ+ individuals tend to be at an increased risk of sexual abuse (SA; Atteberry-Ash et al., 2020) compared to heterosexual cisgender individuals. Disclosure via internet platforms has been shown to hold important benefits for SA survivors (Andalibi et al., 2018). Such platforms are a vital resource for SA survivors as they are sometimes the only source of a supportive community for disclosing their abuse stories (Bogen et al., 2018). One example of this is Torenu, an Israeli Instagram page aimed at providing a platform for members of the LGBTQ+ community to share incidents of sexual abuse and harassment they have experienced. In light of this, the purpose of the current study is to explore the SA disclosures of individuals in the Israeli LGBTQ+ community on the Torenu Instagram page (Torenu, n.d.).

### 1.1. Sexual Abuse in the LGBTQ+ Community

The literature addressing SA in the LGBTQ+ community has identified several key characteristics. In particular, it has shown that LGBTQ+ individuals experience unwanted sexual experiences up to two times more than those in the heterosexual population (Atteberry-Ash et al., 2020). For example, LGBTQ+ students, in general, are more likely to experience sexual victimization and harassment (Tilley et al., 2020) at a rate of almost four times that of their heterosexual peers (DeKeseredy et al., 2017). Regarding subgroups within the LGBTQ+ community, an epidemiological study found increased rates of sexual assault among lesbian and bisexual women compared to heterosexual women (Canan et al., 2021). Sexual assault is also highly prevalent among transgender individuals. According to the U.S. Transgender Survey, 47% of respondents had experienced sexual assault, and 10% were sexually assaulted within the past year alone (James et al., 2016).

An earlier study examined the relationship between sexual and gender identity and four measures of sexual victimization: (1) touched without consent, (2) attempted penetration without consent, (3) penetration without consent, and (4) sexually abusive relationships. The findings showed that gay and bisexual men and bisexual women were more likely to experience all measures of sexual assault than heterosexual men and women (Johnson et al., 2016). Generational differences, reflected in the varying experiences of individuals in their 20s compared to those in their 40s, are also evident in the types of sexual assault experienced and perceptions of sexual assault. These differences can particularly be seen in the shift from traditional and physical arenas to virtual dating apps (Roberts & Ravn, 2020), leading to new means by which SA may occur, such as the nonconsensual distribution of intimate images or videos (Bonilla et al., 2021). Additionally, there is a noticeable difference in the willingness of younger individuals to learn about sex education that normalizes LGBTQ+ identities compared to previous generations (Brandenburg et al., 2024).

### 1.2. Disclosures of Sexual Abuse by Survivors from the LGBTQ+ Community

The existing academic literature has examined the factors that hinder or facilitate disclosure among SA survivors, highlighting the significant influence of characteristics such as age and gender identity (Alaggia et al., 2019). These factors are relevant to all survivors. Nevertheless, there are unique elements relevant to members of the LGBTQ+ community (Cohen, 2016), among them stigmatization, lack of conformity to a stereotypical presentation of SA, and the “double closet”, referring to the need to conceal both one’s sexual orientation and SA (Edwards et al., 2022).

LGBTQ+ SA survivors tend to experience stigmatization from mainstream society in relation to their multiple marginalized identities, namely, being part of a sexual minority as well as being an SA survivor (Edwards et al., 2022; Koon-Magnin & Schulze, 2019; Smart et al., 2021). For instance, LGBTQ+ survivors’ concern that they will be blamed for being sexually abused is significantly more likely to deter them from disclosing than heterosexual survivors (Richardson et al., 2015). Furthermore, non-conformity to a stereotypical model of SA is another aspect at play regarding SA disclosure in the LGBTQ+ community. For example, legal definitions of rape likely exclude the experiences of sexual minority communities (e.g., non-penetrative SA, same-sex abuse; Koon-Magnin & Schulze, 2019). The third factor of the double closet refers to the threat of “being outed”, both regarding one’s LGBTQ+ identity and SA (Cohen, 2016; Edwards et al., 2022). Thus, survivors, particularly those abused by a perpetrator of the same gender and who are still “in the closet”, may experience threats of being “outed” due to sexual victimization or exploitation (Messinger & Roark, 2018).

### 1.3. The #MeToo Movement and Its Impact on Sexual Abuse Disclosure by Members of the LGBTQ+ Community

Disclosure via various internet platforms has important benefits for SA survivors, as disclosing to someone in person may be difficult and complex, in addition to often being silenced (Alaggia & Wang, 2020; Bogen et al., 2018). Such platforms are a vital resource for survivors as they are sometimes the only source of a supportive community for those disclosing their abuse stories (Bogen et al., 2018). For example, survivors have been found to disclose SA on platforms, such as Twitter, when they experience inadequate in-person support, to seek out relevant information, or in the hope of influencing policy (Bogen et al., 2021). Moreover, Alaggia and Wang (2020) reported that disclosure via #MeToo on Twitter and Reddit provided SA survivors with a meaningful way to disclose and find a collective voice online.

### 1.4. #MeToo in the Israeli LGBTQ+ Community and the Torenu Instagram Page

Recently, there has been a major trend of disclosing SA in the LGBTQ+ community in Israel (Segal, 2021). This trend has been referred to as the Israeli #MeToo for the LGBTQ+ community and was most apparent on the “Torenu” Instagram page (Torenu, n.d.). Torenu, meaning “our turn” in Hebrew, is an Israeli Instagram page aimed at providing a platform for members of the LGBTQ+ community to share incidents of sexual abuse and harassment they have experienced. Since it was created in 2021, more than 60 posts have been shared by community members describing experiences of sexual abuse and harassment occurring within the community (Stern, 2021). It should be noted that Torenu was one of the first significant outlets for public SA disclosure in the Israeli LGBTQ+ community. The page has attracted public and social media interest, leading to extensive media coverage and the disclosures of hundreds of Israeli LGBTQ+ SA survivors on social networks.

Due to SA allegations involving senior leadership figures in the LGBTQ+ community, an independent committee published a report presenting different types (e.g., sex work), spaces (e.g., night clubs), and contexts (e.g., religiosity) of SA in the community. The report also pointed to specific LGBTQ+ groups at greater risk of SA, such as trans people. Most importantly, it emphasized the urgent need to address this phenomenon in the Israeli context (Ben-Or et al., 2022). As there is a lack of scientific knowledge on LGBTQ+ SA experiences in Israel, the current study aims to further the understanding of SA in the Israeli LGBTQ+ community through an analysis of SA testimonies posted on the Torenu Instagram page.

The founder and director of Torenu, Omri Feinstein, is a gay man in his twenties who works in advertising. In interviews, he has described how the phenomenon of sexual abuse and harassment within the various LGBTQ+ communities is extremely widespread, yet it remains almost invisible in community discourse. He posted an open question on his personal Instagram page asking LGBTQ+ individuals to “tell me about cases where you were sexually harassed”. This resulted in numerous testimonies, which he defined as the catalyst for opening an official and dedicated page for this issue. Feinstein further shared how, from the moment the page opened on 8 June 2021, it received numerous testimonies. The page was most active between June and July 2021, continuing into 2022, with a few posts published monthly.

## 2. Materials and Methods

### 2.1. Study Sample

To encompass the variety of SA experiences published on the Torenu Instagram page, all posts from 28 June 2021, until 23 January 2022, were considered for inclusion in this study. This time range was chosen as it represented the time period when the Instagram page was established until the activity of the page began to dwindle. Posts were included that met the following criteria: (1) referred to any form of SA (e.g., touching, kissing, physical clinging, etc.) and (2) a personal testimony of being sexually assaulted. Posts were excluded if they did not include a personal testimony of an SA experience (e.g., posts referring to media articles, invitations to a demonstration in support of survivors, etc.). The posts were collected by the research team and stored in an Excel document on the authors’ private computers. Of the total 96 posts, 31 were excluded. The final sample comprised 65 posts, each describing the experiences of a different individual.

The content of the posts identified that the majority (*n* = 54) described men victimized by men, with two posts involving women as both perpetrators and survivors. The remaining posts did not specify the genders of the perpetrators or survivors. It is important to note that the posts were in Hebrew, which is a gendered language. Therefore, it could be assumed that the people who posted their experiences identified as men when they referred to themselves using masculine grammar and women when using feminine grammar. Further details about the gender identities of the people who posted and perpetrators were unclear, as Feinstein anonymized them prior to publication to protect the confidentiality and anonymity of those who shared their stories. For example, it was unclear whether the posts referred to cisgender or transgender individuals. Furthermore, there was no clear reference to bisexual individuals.

The types of abuse ranged from unwanted touching (*n* = 25), kissing (*n* = 5), and physical clinging (*n* = 4) to forced penetration or rape (*n* = 16). Most testimonies (*n* = 50) described an isolated event of abuse by a perpetrator, while others (*n* = 7) revealed abuse that occurred more than once. At least 15 incidents involved perpetrators who were older than the victims. The majority of the SA incidents were described as physically violent and coercive without the survivor’s consent.

### 2.2. Data Analysis

This study was informed by the principles of Queer Theory (Butler, 2002), which challenges the traditional and normative understandings of sexuality and gender. Therefore, the epistemology of this study relied on taking a critical perspective regarding conventional views of knowledge. A thematic analysis was conducted according to Braun and Clarke’s (2006, 2022) guidelines. The analysis was carried out to identify and analyze the patterns of meaning in the data and to highlight the most salient constellations. The authors used Excel 365 software to facilitate the process of analyzing the data. First, the authors independently read the data multiple times to gain an initial understanding of the public discourse. Second, all posts, comprising between 46 and 240 characters, were coded into initial groupings related to their content to create a conceptual tool to classify, understand, and examine the data. These codes were then developed using an inductive approach (Braun & Clarke, 2022) grounded in the content of the data, focusing on semantic meanings. For example, all posts referring to a perpetrator being significantly older than the victim were grouped together, and those describing SA occurring when the victim was under the influence of drugs were grouped together. Each post was assigned to multiple codes. Next, some codes were removed or revised, and new codes were added after rereading the data and in consultation among the authors.

Third, the codes were placed into potential themes reflecting patterns within the data alongside supporting quotes from the raw data. Some initial codes became main themes, while others became subthemes. For example, the codes “perpetrator as much older”, “perpetrator in a position of authority”, and “perpetrator more sexually experienced” were merged into the theme of “power relations”. The authors discussed the codes, how to arrange them, and decided on the appropriate themes. The themes and subthemes were then reviewed and reclassified as necessary (Strauss & Corbin, 1998). For example, the perceptions related to the SA characteristics were merged into one theme and later reclassified into four subthemes: normalization, numbness of the senses, publicness, and escalation. Finally, the authors collaboratively created a thematic map, and two main themes were agreed upon and named, along with each subtheme.

### 2.3. Trustworthiness

Trustworthiness was an important consideration. Therefore, the following methods were employed. The research team conducted weekly meetings in the initial stages of this study, followed by regular meetings in the subsequent stages. These meetings were held to ensure that the coding and synthesis were unified and systematic throughout this study (Nowell et al., 2017). In addition, the methodology and analysis were reviewed by peers who were SA and qualitative research experts. Furthermore, the authors linked relevant quotes to each interpretation to establish rigor. Illustrative quotes selected by the authors were translated into English by a professional translator. In addition, the data collected for this study are publicly available; however, a sensitive approach was taken during the analysis to protect the privacy of the participants. Although the participants shared their experiences anonymously in a public forum, measures were taken to minimize the risk of identification and to ensure their dignity and privacy were maintained.

Qualitative research involves a high degree of reflexivity (Wadams & Park, 2018). Therefore, it is necessary to acknowledge the researchers’ positionality. All authors are part of a qualitative research group exploring sexuality and SA in the LGBTQ+ community. Two authors identify as part of the LGBTQ+ community, while the other two do not. These positionalities have potential benefits in providing insights and understanding regarding the participants’ words and intentions (Wadams & Park, 2018), such as insider knowledge or objective perspectives. However, such positionalities could also affect the interpretations through prior biases. To mitigate this, each author kept a journal documenting their personal processes. Additionally, many discussions were held to further explore and understand the impact of the researchers’ personal lives on the research (Ben-Ari & Enosh, 2011).

## 3. Results

This study focused on the unique characteristics of SA from the perspectives of LGBTQ+ survivors, as reflected in their disclosures of SA on Torenu, a page on the social media platform Instagram. Two main themes were identified through the analysis of the Torenu Instagram posts. The first theme described the survivors and the perpetrators. The second theme highlighted the characteristics of the abuse, focusing on four key aspects: publicness, normalization, numbness of the senses, and escalation.

### 3.1. The Survivors and Perpetrators

#### 3.1.1. The Survivors

When the survivors presented themselves, their undeniable experiences of victimization stood out, either while being harmed or afterward. In most of the posts, the survivors made it apparent that they were indeed harmed, despite the perpetrator’s manipulations designed to make them feel as if they were willing partners in the sexual acts. The survivors made a clear distinction between themselves and the perpetrators. Such distinctions may be due to the fact that the posts were in response to a request to address SA stories.

In the majority of the posts, the survivors wrote in the first person and stated their age at the time of the abuse. Most described themselves as being in their teens or twenties (e.g., “I was 15”). They then described the perpetrator in relation to themselves regarding differences in age or status (e.g., “He was 40, a famous singer”). In this way, they emphasized their vulnerability and the power imbalances between themselves and the perpetrators. The survivors sometimes added a description of themselves, explaining their high level of vulnerability, relating to factors such as their minority status (e.g., part of a closed religious community), fragile emotional state, or being in a position that required them to obey authority (e.g., an employee). These aspects are further reflected in the following two posts:

I was 14 years old and a student in a high school Yeshiva [Jewish religious school for boys]. I was confused, and I asked the teacher-Rabbi for some good advice. He said he would help me become straight. I only realized it wasn’t innocent treatment when he was arrested. He touched me and sexually assaulted me continuously for four years.

I worked as a salesman in a clothing store, and I was helping a customer. At the end, I came to take his items from the dressing room. While he was getting dressed and handing me the items, he pulled my hand and tried to force me into the dressing room.

The survivors also presented the ways they reacted during the abuse. They described states of freezing, dissociating, resistance, and attempting to escape, as well as the transitions between their responses. Here, a freezing response was described:

I was 20 years old, a combat soldier. I went to the clinic at the base to get a referral to a doctor at a hospital. The doctor ordered me to take off my pants and underwear. I did as he said, and he began to jack me off. I was in shock, but I froze and did not react. I waited for it to be over.

Others shared their responses of resistance and running away:

As soon as I arrived at his place, he grabbed me from behind and pushed up against me with his penis. I had to push him away from me, and I ran away crying.

#### 3.1.2. The Perpetrators

The perpetrators, primarily identified as men, were most often described as violent and coercive, using physical force, manipulations, or threats to carry out the abuse:

We met at his place. We started with oral sex, and then he tried to aggressively force me to have anal sex. I refused. He was a strong man. He had a large dog that was barking in a nearby room. He flipped me over, held both my hands tightly, and said that if I continued to resist, he would release the dog. I disconnected from my body and let him continue.

This post reveals the survivor’s intense and traumatic experience, highlighting the perpetrator’s use of intimidation and threats to commit the abuse. Similar experiences are described in the following post:

She invited me to her place. The atmosphere was not great, but somehow we still ended up in bed. At one point, she got upset about something and I told her I wasn’t into it anymore and that I wanted to leave. “Are you kidding?” she replied, and penetrated me. After I pushed her off of me, I got dressed and left. In the morning I received a message from her saying that she hoped I would not tell anyone that I had not cum.

From this post, we can see the ongoing psychological manipulation and emotional abuse that continued even after the event.

### 3.2. The Characteristics of the Abuse

Four main aspects characterized the abuse: publicness, normalization, numbness of the senses, and escalation.

#### 3.2.1. Publicness

Many posts referred to abuse that occurred in public places where an audience was present. Sometimes the abuse occurred in secrecy, for example, in a washroom at a party, or in ways that were visible to others, like while dancing at a party or in front of other students. Performing the abuse in public places may have allowed the perpetrators the opportunity to treat it as a legitimate, normative, and acceptable act. Sympathetic responses or the participation of others in these spaces, as well as their disregard or lack of response, reinforced the legitimacy of the abuse. Therefore, its publicness was used to the advantage of the perpetrators:

On the bus, an elderly ultra-Orthodox man decided to stroke my groin right next to the driver and asked me if I would lend it to him for the weekend.

When I was 18, I went out to a party for the first time. Someone forced himself on me, even though I made it clear to him several times that I was not interested. The security guard saw and chose not to intervene. It ended up with him forcing me to touch him.

When I became a sergeant in the army, as part of the hazing, I was passed around on the floor in the shower, from one hand to another, humiliated, and at one point, someone pushed a finger covered in soap into me so that it would burn. It was all done in an atmosphere of joking and an “initiation ceremony”.

#### 3.2.2. Normalization

The majority of the posts described how the perpetrators presented the abuse as part of generally accepted norms. They conceptualized their abusive actions as something that should be assumed to occur and related to either sexual intercourse norms in general or the LGBTQ+ community in particular.

“All men want it”, he said when he was drunk, lying on me forcibly and pushing his tongue into my mouth, even though I resisted.

He penetrated me and it hurt. I asked him to stop but he kept going until I pushed him away from me by force. Then he tried to push his cock into my mouth. I refused. “Be a bottom and shut up”, he berated me.

Notably, the conceptualization of “being a bottom” produced legitimacy for abuse, as though being a bottom also meant being passive and lacking choice or will.

#### 3.2.3. Numbness of the Senses Caused by Substances

Many of the posts referred to the use of drugs or alcohol before and during the abuse. These substances were either knowingly consumed by the survivor or given to them without their knowledge. The use of substances not only accompanied the abuse but also shaped it. Sometimes, the effects of the substances on their body or minds meant that the survivors had no memory of the abuse. They only recalled what happened before and after, or they woke up in the midst of an abusive situation. This often made it difficult for them to understand if they had indeed been abused. Nevertheless, these circumstances evoked a strong sense of victimhood, as they were subjected to sexual acts when they could not resist them, as well as feelings of guilt and shame.

I was wasted from alcohol and I threw up, but in someone else’s house. When I collapsed on the sofa, he put his cock in my mouth.

Two years ago, I went to a party with my partner and his friends, and I did MDMA for the first time. I do not remember anything from immediately afterward. Chaos. A mess. I woke up with a guy who was a stranger inside of me. I did not understand who he was. I froze. I asked him to stop and leave, and he said, “You are so beautiful … just relax, try to let go … your partner won’t be angry”.

#### 3.2.4. Escalation

Many posts referred to sexual encounters that, at first, were reciprocal, physically pleasant, and consensual. However, they then became one-sided, unwanted, and abusive when the perpetrator continued despite the survivor’s request to stop. At times, the perpetrators continued the SA through force and threats, causing the survivors to resist, freeze, or disengage:

I was 15. We started touching each other. We were friends. It was nice, until he asked for more and I said no. Then, he went into the room, forced me in, and locked the door.

When I was 16, I had a girlfriend who was two years older than me. The first time she came to sleep at my place she tried to touch me many times, and each time I said “no”, to the point where I had to try to protect myself with my hands. She told me that, with me, she just could not control herself. At one point I did not manage to resist, and it became rape.

Overall, the analysis found that the survivors often presented the perpetrators as being older and having authority, and themselves as helpless in the face of the SA. Sometimes they tried to run away or resist, and sometimes they froze.

## 4. Discussion

This study aimed to shed light on SA perpetrated against individuals in the LGBTQ+ community through an analysis of testimonies of sexual abuse and harassment posted on the Israeli Instagram page, Torenu (n.d.). In this study, two major themes emerged: (1) perpetrators and survivors and (2) characteristics of the abuse.

The analysis indicated that the perpetrators were often older than the survivors, strangers or brief acquaintances, and violent and coercive. Moreover, the findings demonstrated that the survivors presented themselves as vulnerable and were sometimes called a “bottom” in a derogatory way. Some of the literature has suggested the discrimination against “effeminate” gay men and the association of feminine traits with being a “bottom” sexually. This discrimination may be related to framing gay sexual relationships through a traditionally heterosexual and masculine lens (Ravenhill & Visser, 2018; Vytniorgu, 2022).

The findings also showed that the survivors were vulnerable due to power imbalances between themselves and the perpetrators, such as differences in age and status. Accordingly, Foucault (1977) has argued that power is diffused and embedded in social structures, exerted not only through overt coercion but also through norms, discourses, and institutional structures that regulate bodies and identities. An example from the findings, which has also been emphasized in the existing literature, is the presence of perpetrators in religious communities, where figures of authority, such as rabbis or teachers, abuse their positions of power (Child Abuse Royal Commission, 2017). Conversely, some of the previous literature has focused on the positive aspects of age gaps in gay relationships, pointing to the supportive side (Silva, 2023). Nevertheless, it was evident in the posts that power gaps played a role in the survivors’ reactions to the SA, which included freezing, dissociating, resisting, or attempting to escape.

Additionally, as mentioned earlier, most perpetrators were strangers, and the SA often occurred in public areas. These findings could be understood via the arenas where the abuse occurred, namely, dating apps and parties, which are public arenas (virtually and physically) that could lead to encounters with strangers (Rowse et al., 2020). This is in contrast to SA within the heterosexual–cisgender society, which often occurs in private spaces with a familiar perpetrator (Association of Rape Crisis Centers in Israel, 2018).

The analysis demonstrated that the survivors tended not to report the abuse close to the time it happened, which left them alone to deal with the consequences. In some cases, the SA occurred before the disclosure of their sexual orientation and gender identity to those close to them. Such cases have been described as the double closet, referring to the secrets of both the SA and LGBTQ+ identity, which lead to an increased risk of SA (Cohen, 2016; Edwards et al., 2022).

The findings related to the normalization of SA may be explained by traditional stereotypes that portray men as sexually dominant, which are used against gay and bisexual men not only within the gay community but also in broader heterosexual–cisgender society (Beam & Wellman, 2022). Additionally, a study on sexual victimization among queer women found that marginalized individuals who perpetrated SA were influenced by traditional masculinity in an attempt to reclaim power (Bedera & Nordmeyer, 2021). Thus, the boundary between adopting norms and stereotypes concerning roles in SA can be ambiguous.

Normalization is concerning and should be treated as a warning sign for gray areas that are harmful yet considered legitimate. The current findings are similar to a recent report by an independent committee of researchers and professionals in Israel (Ben-Or et al., 2022) that examined the issue of SA in the LGBTQ+ community and, particularly, in social organizations. The committee’s findings indicated that, sometimes, survivors do not know how to identify SA because the event or experience is classified within community norms. This was further intensified when the abuse occurred in specific LGBTQ+ spheres, such as parties or under the influence of substances (Ison et al., 2025). In Israel, the legal protection of those who are part of the LGBTQ+ community is still in its infancy. Consequently, the current study, alongside other related studies, can provide the framework for understanding how Israeli LGBTQ+ people experience SA.

### 4.1. Implications

This study contributes to increasing awareness of SA in the LGBTQ+ community and sheds light on the characteristics of perpetrators and survivors as well as SA. The accumulated knowledge of this study, resulting from the posts on the Torenu Instagram page, can teach us about the uniqueness of SA of individuals in the LGBTQ+ community. Furthermore, the findings may help more cases to be recognized and treated earlier and more effectively. Sharing the experiences of others could also help stop the acceptance of SA as the norm in the LGBTQ+ community. The current study also encourages sensitive discourse on SA in the LGBTQ+ community to reduce the stigmatization among the general public. Additionally, in-person bystander training could foster positive changes in attitudes and behaviors by enhancing awareness of the issue and a sense of responsibility to address it (Mujal et al., 2019). On the macro level, legal protection and severe punishment for sexual crimes perpetrated against individuals belonging to minority sexual orientations and gender identities should be enacted to promote the safety and acknowledgment of the equal position of LGBTQ+ people.

### 4.2. Limitations

The current study has several limitations. First, the sexual orientation and gender identity of the survivors and perpetrators, as well as their ages, were either not referred to or not clearly noted in many of the posts. Therefore, some of this information was missing. Furthermore, the posts did not identify specific LGBTQ+ groups, such as the trans community. In addition, it was not clear which perpetrators in this sample identified as members of the LGBTQ+ community. Second, the length of the posts was short, often no more than 240 characters. Therefore, some details may have been omitted, impeding the understanding of the context of the SA. Third, information regarding Feinstein’s process of choosing and publishing the posts is unknown. Lastly, the Torenu Instagram page asked LGBTQ+ community members to share the incidents of SA they experienced. Thus, it is likely that only those who saw themselves as SA survivors posted testimonies. Future studies should explore cases where the positions of the perpetrators and survivors are less clear or understood.

## 5. Conclusions

The disclosure of SA among members of the LGBTQ+ community through social media in Israel is part of a recent process of disclosure, which lends itself to a discourse that addresses the gray areas, norms, and boundaries between sexuality and SA within the LGBTQ+ community (Ben-Or et al., 2022). Yet, this discourse cannot exist without the benevolent intervention of the general society. Research has shown how teens with an LGBTQ+ identity feel excluded from sex education, if such courses are even offered at their schools (e.g., Gegenfurtner & Gebhardt, 2017; MacAulay et al., 2022). This points to the absence of LGBTQ+ identities in the general sexual discourse, which creates an experience of loneliness and invisibility for those in this community. Furthermore, the conceptualization or understanding of healthy sexuality is challenging as there are no models LGBTQ+ individuals can refer to nor frameworks they could participate in within the general society. Thus, society must take responsibility to encourage discourse on the broad spectrum of sexuality to accept its validity and reduce stigmas related to sexuality within the LGBTQ+ community. This, in turn, may promote disclosure and intervention in SA cases within the LGBTQ+ community via social media and other arenas.

## Data Availability

The data presented in this study are openly available on Instagram at https://www.instagram.com/torenu.lgbt/ (accessed on 1 March 2022).
